# Effectiveness of a multidisciplinary treatment with youth-initiated mentoring for youths with mental health needs from multi-problem families: a quasi-experimental study

**DOI:** 10.1186/s12889-023-17506-6

**Published:** 2024-01-02

**Authors:** Natasha Koper, Yukiko Boin, Hanneke E. Creemers, Levi van Dam, Geert Jan J. M. Stams, Susan Branje

**Affiliations:** 1 Department of Youth and Family, Utrecht University, PO box 80140, Utrecht, 3508TC the Netherlands; 2 Department of Forensic Child and Youth Care Sciences, University of Amsterdam, Amsterdam, the Netherlands; 3 YIM Foundation, Amersfoort, the Netherlands; 4 Levvel youth and family care, Amsterdam, the Netherlands

**Keywords:** Effectiveness, InConnection approach, Multidisciplinary treatment, Multi-problem families, Quasi-experimental trial, Youth-initiated mentoring

## Abstract

**Background:**

Children from multi-problem families have an increased risk for experiencing mental health problems. These families face problems in several domains that are often found to be chronic and intergenerational. Yet, the effects of mental health care for youths from multi-problem families are small at best, urging research on new treatment programs. The InConnection approach is an integrated care program to improve resilience of youths with mental health needs from multi-problem families by connecting professional expertise from multiple disciplines with the informal social network of the youth. Youths are asked to nominate a youth-initiated mentor (YIM) from the supportive adults in their network.

**Methods:**

This quasi-experimental study compared the effectiveness of the InConnection approach to treatment as usual in a sample of 107 families (*n* = 66 intervention group, *n* = 41 control group) with *n* = 115 youths receiving treatment (cases). Youths (*n* = 102 reports, *M*_age_ = 15.59 years), parents (*n* = 85 reports) and case managers (*n* = 107 reports) responded to questionnaires four times over 15 months. Using these data, we measured youth resilience as the primary outcome, seven secondary outcomes, and three intermediate outcomes.

**Results:**

Latent growth models showed only one significant change in outcomes over time across conditions, namely a decrease in case manager-reported child unsafety, and only two condition effects, which were both parent-reported. Parents in the InConnection group reported improvements over time in youth’s emotional and behavioral problems and their own positive parenting, whereas control parents reported no changes (*p*s ≤ 0.013).

**Discussion:**

The treatment conditions were not effective in improving most of the youth and parental outcomes over time, except for child safety reported by the case manager. The InConnection approach only outperformed care as usual on two parent-reported outcomes. Future research should examine for whom and under what circumstances the InConnection approach works more convincingly.

**Trial registration:**

Netherlands Trial Register NL7565. Retrospectively registered on 05/03/2019.

**Supplementary Information:**

The online version contains supplementary material available at 10.1186/s12889-023-17506-6.

Multi-problem families face several problems in multiple domains, such as family functioning, mental health, financial situation and social network (e.g., conflicts with others), which are often chronic and intergenerational [1, 2]. Such problems may place the development of children growing up in those families at risk [3]: Children in multi-problem families experience more internalizing and externalizing behavior problems and a lower quality of life compared to children in the general population [1]. Not surprisingly, both parents and children of multi-problem families receive more mental health care, have a longer history of care, and receive more intensive care, such as out-of-home placements, than parents and children in the general population [1].

Given the severe and chronic difficulties faced by multi-problem families, effective care approaches are urgently needed. Yet, studies examining the effectiveness of care for youths experiencing multiple problems and youths growing up in multi-problem families show small effects at best [4–7], suggesting that we need better evidence-based forms of care. That said, youth mentoring has positive effects for youths of different risk levels, including youths of multi-problem families, across a broad range of outcomes [8–10]. The current study examined the effectiveness and mediating mechanisms of an innovative multidisciplinary systemic treatment including mentoring for youths of multi-problem families. The theoretical background and design of this effectiveness trial have previously been reported in a study protocol [11].

## Treatment as usual for multi-problem families

Treatment for multi-problem families is commonly systemic or family-based. These treatment programs generally provide customized care in multiple domains and strive to actively involve the family system in decision making [12]. Given the complexity of problems, multi-problem families often receive support from various care providers. This may result in fragmentation of care, hampered coordination between professionals and institutions, and single solutions for complex problems [13–15]. To avoid this, treatment approaches have been developed in which various forms of care can be integrated and coordinated by a case manager or family guardian who functions as the link between the family and professional care services. Examples are the ‘Wraparound care’ model in the United States [16], the ‘Troubled Families’ program in the United Kingdom [17], and the ‘One family, one plan’ policy in the Netherlands [18].

These approaches and policies integrate *formal* care systems, that is, care provided by organizations in formal settings (e.g., health care and social services). Very few integrate formal with *informal* care systems [19, 20], that is, a family’s informal social network including family, friends and other social groups. Yet, involving the social network is thought to contribute to the effectiveness of care [21], as strong social support networks are linked to higher levels of resilience, or successful adaption in face of adversity [22, 23]. Thus, treatment programs could potentially be enhanced by promoting the coordination between formal and informal support [15] and using the full potential of families’ support systems.

## The InConnection approach

The InConnection approach is an innovative, multidisciplinary treatment program for youths of multi-problem families, which integrates formal and informal care to increase resilience and to prevent out-of-home placements. It aims to increase effectiveness of care compared to treatment as usual in two ways. First, the approach differs from treatment as usual for multi-problem families [24] in that it provides care by a multidisciplinary team consisting of professionals specialized in youth and family care, psychiatry, addiction care, and care for people with mild intellectual disabilities. The InConnection approach thus not only includes a case manager who coordinates care from different organizations or types of expertise, but also brings the different types of expertise and care together within one approach and team. This approach offers families direct access to a wide range of specialized treatment possibilities, depending on the family’s needs [25]. Examples are youth-focused treatments, such as cognitive behavioral therapy and psychomotor therapy; caregiver and family-focused treatments, such as parent training and trauma therapy; and multisystem treatments, such as multisystemic therapy. Despite the different treatment forms, families experience continuity of care as treatments are coherently organized to meet the family’s needs and preferences [26].

Second, InConnection utilizes the potential of the informal network by actively involving a youth-initiated mentor (YIM) from the youth’s social network [25, 27]. In the first phase of treatment, youths nominate an informal mentor as their YIM from the supportive adults within their social network. The YIM is a confidant and spokesperson for the youth, and a partner for parents and professionals [28]. During treatment, all members of the client system, including the YIM, actively participate in the decision-making process by giving their perspectives on desired treatment goals and contributing to reaching these goals [25]. The active participation of the client system stimulated by the InConnection approach is assumed to make the approach more client-focused and strength-based than care as usual. Moreover, rather than directly addressing the problems in a family, the InConnection case manager guides and facilitates a collaborative process that contributes to sustainable improvements [24, 25].

## Effectiveness of the InConnection approach

The potential of integrated care and (youth-initiated) mentoring to enhance treatment effectiveness has been empirically supported. Integrating (mental) health care is considered to improve treatment effects and efficiency, quality of life, and client satisfaction in healthcare in general [26], and treatment for multi-problem families, specifically [29]. Furthermore, YIM programs significantly improve youth functioning in different domains, such as academic and vocational functioning, social-emotional functioning and psychosocial problems for youths with different risk levels [10, 30]. In addition, preliminary positive results of the InConnection approach have been found. In two studies with a total of 138 youths of multi-problem families, approximately 80–90% of youths continued to receive outpatient treatment only, despite a prior indication for out-of-home placement [27, 31]. Yet, both studies have methodological limitations, such as the lack of a control group [31] and a retrospective quasi-experimental case-file-analysis design without measures of youth adaptivity [27]. Therefore, the current study examined the treatment effects and mediators of the InConnection approach in a more rigorous, quasi-experimental design [11].

## Mediators of treatment effects

Treatment mediators or intermediate outcomes determine how treatments work [32]. Three potential mediators are assumed to explain how the InConnection approach improves youth resilience and well-being: social resourcefulness, shared decision making and treatment motivation.

The experience of a supportive relationship with a YIM may increase youth’s social resourcefulness [24], or the ability to seek help and support from the social network. It is suggested that the positive relationship with a YIM provides a safe context for youths to practice and develop their relationship skills, allowing youths to benefit more from the social ties within their network [24]. Indeed, higher quality mentoring relationships are associated with improved relationships with other adults [33, 34]. Moreover, in a qualitative study [35], youths reported they felt more comfortable seeking help after participation in a school-based mentoring program, suggesting a link between mentoring relationships and social resourcefulness. Social resourcefulness was, in turn, found to be related to positive treatment outcomes in school-based settings, such as increased self-esteem, prosocial behaviors, and reductions in misconduct [33, 34]. Thus, we expect that InConnection is more effective in promoting positive youth outcomes such as resilience and well-being than other programs, due to increased social resourcefulness associated with involving a YIM.

Integrated care and the collaboration with a YIM may increase shared-decision making with the client system [24, 29]. Shared-decision making means that goals are set in collaboration with the client system (and its social network), which is thought to result into personal goals that are set for autonomous reasons [24]. Having personal or self-concordant goals has been associated with successful goal progress and achievement [36], suggesting that shared-decision making may increase treatment effectiveness. As integrated care requires a dynamic treatment plan that changes according to clients’ changing needs [29], InConnection actively involves the client system including the YIM in the treatment process and the development and evaluation of the treatment plan [24]. Thus, we expect that shared-decision making serves as a mediator of care effectiveness.

The InConnection approach may also contribute to treatment effectiveness through enhanced treatment motivation. It is long known that treatment motivation is an important factor for treatment effectiveness [37, 38]. YIMs encourage youths to participate in treatment and achieve challenging treatment goals [39]. Moreover, YIM-assisted care may support youth’s sense of autonomy, competence and relatedness which are necessary ingredients for motivation [40]. Youths are encouraged to *autonomously* choose a YIM and participate in shared-decision making, therefore strengthening their sense of *competence* to choose what is right for them [24]. The positioning of a YIM increases the *relatedness* with a supportive Fig. [41] and others [33, 34]. Thus, we expect that youths are more motivated to engage in a treatment program involving a YIM, which may subsequently improve treatment effects.

## Current study

In conclusion, the InConnection approach is a promising treatment for youths of multi-problem families, but its effectiveness in comparison to treatment as usual and potentially important mediators have not been investigated yet in a controlled, prospective multi-informant study. This knowledge is assumed to be essential for expanding evidence-based forms of care. Therefore, the current study tested the effectiveness of InConnection in a quasi-experimental design [11]. We expected that InConnection was more effective than care as usual in promoting youth resilience (*primary outcome*), youth mental health, parent-child relationship quality, and parental functioning; and in reducing the risk of child unsafety and the occurrence of out-of-home placements (*secondary outcomes*). Moreover, we hypothesized that InConnection was more effective in increasing social resourcefulness, treatment motivation, and shared decision making (*intermediate outcomes*), and that these intermediate outcomes would mediate the treatment effects.

## Methods

### Design and procedure

The effectiveness of the InConnection approach was examined in a quasi-experimental trial with two conditions: the InConnection approach and treatment as usual. Allocation to care programs was non-random, as it depended on the availability of care within a specific program (sometimes programs had a waiting list and clients were therefore allocated to the other form of care) and the client’s preference for the content and methods of one care program over the other.

Families with multiple, complex problems registered for intensive youth care at one of the five participating organizations were eligible to participate. These organizations were situated in urban areas in the Netherlands, and were selected because they offer a variety of youth and family care for multi-problem families. Each organization offered the InConnection approach and one or more other approaches for systemic outpatient care (treatment as usual). Upon registration for one of the treatment modalities, families were approached for participation in this study if: (1) families consisted of at least one youth aged 10 to 23 years; (2) families experienced problems such as school drop-out, divorce, trauma, antisocial behavior, and substance use that are considered complex, multiple and severe, and/or previous treatments had not yielded the intended effects, and/or youth had an indication for an out-of-home placement; (3) families had sufficient Dutch proficiency.

To assess changes in outcomes during treatment, four multi-informant (youth, parent, YIM, and case manager) assessments using Dutch-language questionnaires were conducted between January 2019 and January 2022: (1) at the start of treatment (T1); (2) after three months (T2); (3) after nine months (T3); and (4) after 15 months (T4). At the first assessment, youth, parents and YIMs completed questionnaires at a chosen location, often at home, in the presence of a researcher who assisted participants in answering the questions. If the participant was 16 years or older and did not experience problems in answering the questions, the subsequent assessments were completed online. To comply with the measures against the coronavirus taken by the Dutch government, we replaced home visits by phone and video calls from March 2020 onwards. Case managers individually completed online questionnaires at all assessments. Each assessment took approximately 30 min to complete. Participants gave active informed consent for their own participation. For youths under the age of 16, active informed consent for their participation was also received from one parent or legal guardian. Participants received a financial reward of €50 for completion of the four questionnaire assessments. This trial has been approved by the ethical review board of the Faculty of Social and Behavioral Sciences of Utrecht University (FETC-18-093), and is registered at the Netherlands Trial Register (NL7565; for protocol, see 11).

### Participants

**Fig. 1:**
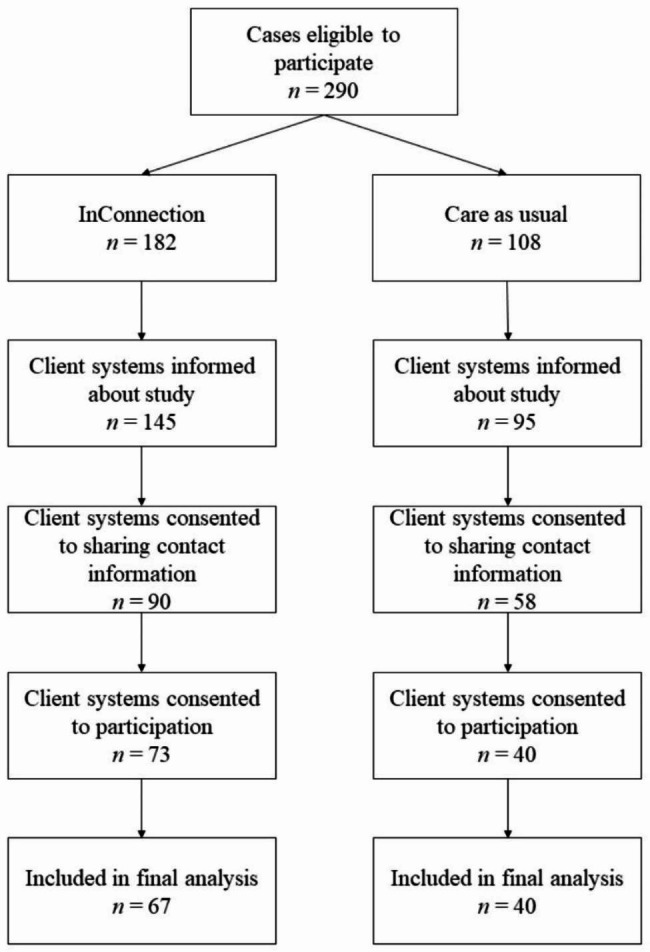
Participant flow per treatment condition

At baseline, the current study included a sample of 107 families of which 66 (62.7%) were in the intervention group and 41 (38.3%) were in the control group (see Fig. 1 for the participant flow). In these families, there were 115 youths receiving treatment (cases). We were able to recruit participants for the intervention group from all five organizations for youth and family care, but two organizations failed to deliver participants for the control group (in one organization because there was no suitable control group, and in the other organization because none of the families wanted to participate). Families started treatment between December 18th, 2018 and September 2nd, 2020. Unexpectedly, the allocated treatment continued after the final assessment (i.e., 15 months after starting treatment) in most cases (66.0%), which did not differ between conditions, χ^2^ [1] = 0.61, *p* = .434. The average duration of completed treatments was 298 days (*SD* = 133.99, range = 40–574), which also did not differ between conditions, *t* [36] = 0.72, *p* = .476. Although we aimed to collect data among youth, parents and case managers for each family, only one person per family needed to participate. Hence, different compositions of informants were possible.

In total, 102 youths (46.1% female), 85 parents (78.8% female), and 58 case managers (70.7% female) reporting on 107 youths participated in the study. Of the youths, 70 (68.6%) were in the intervention group. Youths were on average 15.59 years old (*SD* = 1.73; range = 10.47–18.14) at the start of treatment. Most youths were born in the Netherlands (87.3%) and primarily identified as Dutch (70.6%). At the first measurement occasion, 89.2% of youths attended school, and their living situation was diverse: 47.1% of youths lived with (one of) their parents, 5.9% lived in a foster family, 32.4% lived in an institution, 2.9% lived independently, and 7.8% lived elsewhere (e.g., with family members). Most youths (64%) had experienced an out-of-home placements before starting treatment (range = 1–6 or more).

Fifty-four parents (63.5%) were in the intervention group. At baseline, parents were on average 46.58 (*SD* = 7.23; range = 28.99–64.05) years old. Most parents were born in the Netherlands (80%) and primarily identified as Dutch (88%). More than half of parents completed tertiary education (54%), of which most completed higher education (67%). Other parents completed only primary education (2%), secondary education (27%), a different education (8%), or no education at all (6%). Marital status of parents was diverse: 31% of parents was married, 16% lived together, 27% was divorced, 23% has never been married, and 3% was widowed. See Table 1 for more detailed demographics, including demographics per condition.

**Table 1:** Demographic characteristics of participants and statistical differences between Conditions

	Total		InConnection		Control		Log. regr.
	*n*	%	*n*	%	*n*	%	*p*
Youth	102		70		32		
Gender: Female	47	46.1%	35	50.0%	12	37.5%	0.242
Ethnic identity: Dutch	72	70.6%	50	71.4%	22	68.8%	0.728
School: Yes	91	89.2%	65	92.9%	26	81.3%	0.431
Education level: None/primary	6	5.9%	4	5.7%	2	6.3%	0.112
Education level: Secondary	81	79.4%	60	85.7%	21	65.6%	
Education level: Tertiary	4	3.9%	1	1.4%	3	9.4%	
Liv. sit.: Both parents	19	18.6%	11	15.7%	8	25.0%	0.268
Liv. sit.: Alternately both parents	2	2.0%	2	2.9%	0	0.0%	
Liv. sit.: Mother	22	21.6%	18	25.7%	4	12.5%	
Liv. sit.: Father	5	4.9%	4	5.7%	1	3.1%	
Liv. sit.: Foster care	6	5.9%	5	7.1%	1	3.1%	
Liv. sit.: Professional care	33	32.4%	20	28.6%	13	40.6%	
Liv. sit.: Independently	3	2.9%	1	1.4%	2	6.3%	
Liv. sit.: Other (e.g. with family)	8	7.8	8	11.4%	0	0.0%	
Parents	85		54		31		
Gender: Female	67	78.8%	42	77.8%	25	80.6%	0.756
Ethnic identity: Dutch	75	88.2%	50	92.6%	25	80.6%	0.064
Education level: None/primary	7	8.2%	6	11.1%	1	3.2%	0.411
Education level: Secondary	23	27.1%	12	22.2%	11	35.5%	
Education level: Vocational	15	17.6%	8	14.8%	7	22.6%	
Education level: Higher	31	36.5%	22	40.7%	9	29.0%	
Income: Lowest 10%^1^	52	61.2%	38	70.4%	14	45.2%	0.071
Biological parent: Yes	72	84.7%	46	85.2%	26	83.9%	0.998
Marital status: With partner	39	45.9%	24	44.4%	15	48.4%	0.783
Living with children: Yes	74	87.1%	47	87.0%	27	87.1%	0.829

*Note.* Log. regr. = Logistic regression; Liv. sit. = Living situation. ^1^ The number of people in the lowest 10% of Dutch adults [40]

### Condition differences in demographic characteristics

To determine the demographic equivalence between conditions, we used logistic regressions to examine whether youths and parents in the intervention group differed from those in the control group. As shown in Table 1, we found no demographic differences between the conditions for youths or parents.

Initially, we aimed to include 300 families with a 3:1 ratio to allow for propensity score matching [11]. Since the final sample eligible for analyses turned out to be less than half its aim, we chose not to apply this strategy as this would decrease our sample size even further, making it inappropriate for the analyses planned. This decision was justified by the results indicating no demographic differences between the treatment conditions.

### Missing data

All 102 youths (100.0%) completed the first measurement occasion, 76 (74.5%) completed the second and third measurements, and 74 (72.5%) completed the fourth measurement. Of the 85 parents, 84 (98.8%) completed the first measurement, 60 (70.6%) completed the second measurement, 72 (84.7%) completed the third measurement, and 66 (77.6%) completed the fourth measurement. Case managers reported about 107 youths (100.0%) at the first measurement occasion, about 76 youths (71.0%) at the second and third measurements, and about 57 youths (53.5%) at the fourth measurement. Overall, non-completion was 46.1% for youths, 40.0% for parents, and 62.6% for case managers, indicating that these informants did not complete all four waves. Non-completion was highest for case managers because they were only invited to participate if the youth was still receiving the allocated treatment. If only considering the assessments in which case managers were invited to participate, non-completion among case managers was 36.5%.

Logistic regressions were used to examine differences between participants who completed all waves (completers) and participants who did not (non-completers) on both demographic variables (i.e., organization, condition, and gender for all informants; ethnic identity, living situation, and education level for youths and parents; going to school for youths only; and relationship type, income, and marital status for parents only) as well as study variables at the first measurement occasion. Regarding youths and case managers, the analyses revealed that completers and non-completers did not significantly differ on demographic variables nor study variables, *p*s > 0.136, and *p*s > 0.593, respectively. Regarding parents, completers and non-completers significantly differed on gender, *p* = .044, and parent-reported youth emotional and behavioral problems. Mothers, and parents of children with more problems were more likely to complete all waves. Other variables did not significantly predict completion for parents (*p*s > 0.232).

Missing data of study variables were also analyzed on item level. Little’s Missing Completely at Random (MCAR) test [42, 43] showed that data were missing completely at random (*p* = 1.000 across informants). Moreover, we found a normed chi-square (χ^2^/*df*) of 0.05 for youths, 0.24 for parents, and 0.10 for case managers, suggesting a good fit between the sample scores with and without imputation [44]. Hence, all participants were included in the analyses to allow all available data to be used.

### Conditions

#### InConnection approach

The InConnection approach is a multidisciplinary systemic outpatient alternative to out-of-home care for youths of multi-problem families. Treatment consists of four phases: (1) *who*, (2) *what*, (3) *how*, and (4) *adaptivity* [25]. In contrast to most other treatment programs, the InConnection team does not start with an analysis of problems. Instead, in the *who* phase, the case manager opens the conversation on the value of a YIM and its implications for the family and the professional. The case manager explains that a YIM is someone who is trusted by the youth, someone they can go to for support or advice, and/or someone who inspires them. Youths are asked to think about who could be this person for them. If necessary, the case manager provides more support in identifying a potential YIM, for example by making a social network map. Once youths have identified a potential YIM, this person is nominated by the youth and invited for a meeting with the case manager. The case manager explains what the positioning of a YIM means. If the YIM accepts the position as YIM, all parties meet to discuss issues of confidentiality, privacy, contact frequency, boundaries, and a worst-case scenario, which are laid down in a plan of action. The YIM is officially installed when all parties have signed the plan of action [25]. The duration of this phase is on average one month.

In the *what* phase, all parties give their opinion on what they would like to see changed. Case managers motivate youth, parents, and YIM to discuss the ideal situation. The case manager uses this information to develop a problem analysis and potential solutions. In the *how* phase, all parties work together on formulating a plan of action based on the input from the previous phase. The plan of action documents the treatment goals and the support offered by professionals (e.g., specialized treatment) and the informal network. This plan of action is executed in this phase and evaluated with all parties every two months. The final *adaptivity* phase starts when treatment goals have been met and/or all parties feel that the current professional support is no longer needed. The case manager poses several questions to the youth, parents, and YIM, such as ‘what changes when professional support ends?’ and ‘what happens to the position of the YIM?’. Once all parties agree on how the family will proceed without professional support, the treatment is concluded [25].

As treatment is tailored to the needs of a family, the treatment varies in duration and content. That is, for youths with more complex needs, the treatment may take 12 months or more, whereas for others the treatment may only take 6 months. To tailor the content to the family’s needs, the treatment teams consist of professionals with different types of expertise: youth and family care, psychiatry, addiction care, and care for people with mild intellectual disabilities. These professionals are trained in delivering the treatment according to the InConnection approach to enhance adherence to the guidelines. The number and combination of treatment techniques used differ across families. A few examples: youths with addiction problems can be offered specialized addiction care; parents who experienced trauma can be offered specialized trauma therapy; and families that experience interpersonal conflicts can be offered systemic counselling [25].

##### Treatment fidelity

InConnection case managers completed a questionnaire concerning treatment fidelity at each measurement occasion. The questionnaire was developed for the purpose of this study, and consists of 13 items reflecting the steps in the phases of the InConnection approach (i.e., five items for the *who* phase, two for the *what* phase, and three each for the *how* and *adaptivity* phases). An example item is: “We have described the cooperation agreements between the family, YIM and me as professional”.

On average, case managers reported to have successfully completed about half of the steps (*M* = 7.49, *SD* = 4.54, range = 0–13) throughout treatment. Treatment fidelity varied greatly between cases. In 5 cases (7.0%), case managers did not complete any of the steps. In 34 cases (47.8%), at least three quarters of steps were performed, of which in 14 cases (19.7%), all steps were performed.

#### Care as usual

Care as usual included different outpatient treatment programs for youths of multi-problem families. All selected treatment programs are multi-modal systemic family care programs, such as versions of (intensive) family preservation programs. Team members collaborate with other professionals involved in the family (both from within the same organization and from other organizations). Families could thus be enrolled in several treatment programs at the same time. The average duration of the treatment programs is similar to the InConnection approach: approximately six to 12 months. Short-term interventions, such as crisis interventions, were not included.

#### Manipulation check

To test whether the condition manipulation was successful, case managers reported on the implemented treatment characteristics using the Dutch Taxonomy of Interventions for Families with Multiple Problems (TIFMP), which is developed to register techniques used in the treatment of multi-problem families [45, 46]. The TIFMP included 53 techniques divided over eight domains: (A) assessment and organization of information; (B) planning and evaluation; (C) working on change; (D) teaching parenting skills; (E) task support; (F) activation of the social network; (G) activation of the professional network; and (H) maintaining the collaboration. Case managers indicated whether a technique was used in the period between assessments. If relevant, case managers indicated to whom the technique was directed (youth, parent, family, or environment). The TIFMP was developed and tested in the Netherlands and showed sufficient interrater reliability [46].

Logistic regression analyses revealed that InConnection did not differ from care as usual in the use of techniques from the domain activation of the social network, *p* = .928. However, when looking at whom techniques were targeted to across all domains, intervention techniques used in InConnection were directed at the environment (i.e., social network) more often compared to the control condition (*p* = .010). Reports from youths revealed that 81.4% of youths from the intervention group positioned a YIM, similar to previous research [27, 31].

### Measurements

#### Primary outcome: youth resilience

Resilience of youths was measured by the self-reported Child and Youth Resilience Measure – Short form (CYRM-12), which consists of 12 items [47, 48]. The CYRM-12 assesses the individual, relational, communal and cultural resources available to individuals that may sustain their resilience. Items (e.g., “I have people I look up to”) were rated on a 5-point scale from 1 = *does not describe me at all* to 5 = *describes me a lot*. Higher scores reflect higher levels of resilience. The CYRM-12 showed sufficient content validity to be used as a cross-cultural screener of resilience, and internal consistency was satisfactory in the original Canadian sample [47] and a Dutch sample [49]. In the current sample, internal consistency was satisfactory to good across measurement occasions, α = 0.73–0.84.

#### Secondary outcomes

##### Well-being

Youth and parental well-being was measured using the self-reported World Health Organization Well-Being Index (WHO-5) [50]. Youths and parents rated 5 items (e.g., “I have felt calm and relaxed”) on a 6-point scale from 0 = *none of the time* to 5 = *all the time*. Higher scores reflect higher levels of well-being. The measure is deemed appropriate for cross-cultural screening purposes and to be used in clinical trials [51]. The internal consistency and validity were satisfactory in a variety of samples [51], including a Dutch sample [52]. Internal consistencies were good to excellent across measurement occasions, α = 0.86–0.91 for youth, and α = 0.82–0.91 for parents.

##### Youth emotional and behavioral problems

Youth emotional and behavioral problems were measured using the multi-informant Brief Problems Monitor (BPM). The BPM is the abbreviated version of the Child Behavior Checklist and monitors children’s emotional and behavioral functioning [53, 54]. Youths filled out the self-report version (BPM-Y) and parents filled out the parent version (BPM-P). Both versions consist of 19 items (e.g., “I argue a lot” and “Argues a lot”), which were rated on a 3-point scale from 0 = *not true* to 2 = *very true*. Higher scores reflect more problems. Psychometric properties of the BPM-Y [55] and BPM-P [53, 55] were adequate in American and Norwegian samples: Internal consistency was high and validity was satisfactory. The internal consistencies were good at all measurement occasions, α = 0.83–0.88 for youth, and α = 0.86–0.89 for parents.

##### Risk of child unsafety

Risk of child unsafety was measured using the Actuarial Risk Assessment Tool for Protection of Juveniles (ARIJ), a Dutch assessment tool for professionals to assess the future risk of unsafety of children and youths [56]. Case managers rated 32 items on a 3-point scale with 1 = *yes*, 2 = *no*, and ?=*unknown*. (The item “young child, < 5 years old” of the original ARIJ has been excluded in this study, as youths participating in our study are 10 years or older.) We created sum scores of the 12 dynamic items (e.g., “Concerns about parenting and care: Protection and safety”) to assess the risk of child unsafety across measurement occasions. Higher scores reflect a higher risk of child unsafety. The ARIJ was developed and tested in the Dutch context, and has adequate interrater and intrarater reliability [57]. In the current sample, the internal consistency was satisfactory to good across measurement occasions, α = 0.73–0.85.

##### Out-of-home placements

Youths reported on whether an out-of-home placement took place during the study at the second, third and fourth assessment (yes or no).

##### Parent-child relationship quality

Parent-child relationship quality was measured using the Psychological Availability and Reliance on Adult (PARA), which is designed to measure relationship quality in asymmetrical relationships from an attachment perspective. It measures three aspects of the relationship: availability, reliance, and affective bond [58, 59]. Youths reported on the relationship with mothers and fathers separately. Parents individually reported on the relationship with their child. Three items of the original affectional bond scale have been deleted, as they were not deemed appropriate for the parent-child relationship (e.g., “You dread knowing you may have another [father/mother] in the future”), resulting in a 16-item scale. Items (e.g., “My parent is warm and understanding” and “I am warm and understanding”) were rated on a 4-point scale from 1 = *disagree* to 4 = *agree*. Higher scores reflect higher levels of parent-child relationship quality. Internal consistency and validity were satisfactory for most scales in a Dutch sample [58]. The internal consistencies were good to excellent at all measurement occasions, α = 0.81–0.99 for youth, and α = 0.85–0.90 for parents.

##### Parental resilience

Parental resilience was measured with the self-reported Adult Resilience Measure – Short form (ARM-12) consisting of 12 items [60]. The ARM-12 is an adapted version of the CYRM-12 [47] for use with adults. In contrast to the CYRM-12, psychometric properties of the ARM-12 have not been examined yet. In the current sample, the internal consistency was satisfactory to good across measurement occasions, α = 0.78–0.86.

##### Parental empowerment

Parental empowerment was measured using the Family scale of the self-reported Family Empowerment Scale (FES), which measures empowerment in parenting situations in families with children who have emotional, behavioral or mental disorders [61]. Parents rated 12 items (e.g., “When dealing with my child, I focus on the good things as well as the problems”) on a 5-point scale from 1 = *never* to 5 = *always*. Higher scores reflect greater empowerment. Validity of the Family scale was good in American [61, 62] and Dutch [63] samples. The internal consistency has only been examined in an American sample, and was excellent [62]. In the current sample, the internal consistency was good to excellent across measurement occasions, α = 0.89–0.92.

##### Parenting behavior

Parenting behaviors were measured using the self-reported Alabama Parenting Questionnaire – Short form (APQ-9). The APQ-9 measures three main parenting practices in response to child behavioral problems: positive parenting, inconsistent discipline, and poor supervision [64]. Parents reported their parenting behavior using the 9 items (e.g., “You praise your child if he/she behaves well”) that were rated on a 5-point scale from 1 = *never* to 5 = *always*. Higher scores reflect higher levels of parenting practices in a certain domain. Validity of the APQ-9 was good, but the internal consistency was low in an Australian sample [64]. Yet, a low internal consistency is not necessarily problematic when the purpose is to measure a broad concept using few items, like in the APQ-9. Internal consistency of the extended APQ were low to good in a Dutch sample [65]. Similarly, the internal consistencies were low to excellent across measurement occasions in the current sample, α = 0.83–0.92 for positive parenting, α = 0.65–0.77 for inconsistent discipline, and α = 0.51–0.67 for poor supervision. We dropped one item of the poor supervision scale as it is considered outdated and parents expressed issues with the item (“Your child fails to leave a note or to let you know where he/she is going”). Deletion of this item increased internal consistency to α = 0.62–0.79.

### Intermediate outcomes

#### Social resourcefulness

Youths’ level of social resourcefulness was assessed using the subscale Seeking Social Support of the Dutch questionnaire Utrecht Coping List (UCL). This subscale measures the extent to which youths seek comfort and understanding from others, tell someone about their concerns or ask for help [66]. Youths rated the 6 items (e.g., “Share your worries with someone”) on a 4-point scale from 1 = *rarely or never* to 4 = *very often*. Higher scores reflect more social resourcefulness. The internal consistency and validity of the UCL were good in a Dutch sample [66]. In the current sample, the internal consistencies were good across measurement occasions, α = 0.84–0.89.

#### Shared-decision making

Shared-decision making was measured using the second and third of the Session Rating Scale (SRS), which is a brief four-item measure of therapeutic alliance. These items tap into agreement on the treatment goals and treatment tasks [67]. Youths and parents rated the items on a continuous scale of 10 cm, where the left side indicates a more negative response (e.g., “We did not work on or talk about what I wanted to work on and talk about”) and the right side indicates a more positive response (e.g., “We worked on and talked about what I wanted to work on and talk about”). Thus, higher scores reflect higher levels of shared-decision making. The internal consistency and validity of the SRS including all four items were satisfactory to good in American [67] and Dutch [68] samples. In the current sample, the internal consistencies were good to excellent across measurement occasions, α = 0.88–0.91 for youths and α = 0.82–0.94 for parents.

#### Treatment motivation

Youths’ treatment motivation was assessed using the subscale Motivation to Engage in the Treatment of the self-reported Treatment Motivation Scales for Forensic Outpatient Treatment (TMS-F) [69]. Youths rated the 16 items (e.g., “If I saw little change in my life, I would end the treatment”) on a 5-point scale from 1 = *strongly disagree* to 5 = *strongly agree*. Higher scores reflect greater treatment motivation. Internal consistency and validity were satisfactory in a Dutch adult sample [69]. Psychometric properties have not yet been studied in youth samples. In the current sample, the internal consistency was good to excellent across measurement occasions, α = 0.83–0.94.

### Statistical analyses

Descriptive statistics were obtained through SPSS version 26 to gain insight in the means, standard deviations and correlations among the variables. All other analyses were performed in Mplus 8.7 [70]. Data were analyzed following the intention-to-treat principle, meaning that participants were grouped according to their allocated treatment, regardless of whether treatment was completed or not. Because missing data were missing completely at random, the default setting in Mplus for handling missing data (i.e., full information maximum likelihood) was used. Full information maximum likelihood (FIML) is a gold standard method to handle missing data in structural equation modeling, which incorporates all available information from observed data, including cases with missings, to maximize the likelihood function. FIML ensures that the information from cases with complete data and those with missing data is appropriately combined to estimate parameters accurately [71, 72]. We performed multilevel analyses to account for the nested structure of our data, thus providing unbiased estimates. More specifically, three-level models were examined in which measurement occasions (Level 1) were nested within participants (Level 2), and participants were nested in families (Level 3). Change across time in the outcomes was assessed with latent growth models.

The fit of the models was evaluated using the following cutoff scores [73]. First, for the comparative fit index (CFI), values ≥ 0.90 would indicate acceptable fit and values ≥ 0.95 would indicate good fit. Second, for the root-mean-square error of approximation (RMSEA) and the standardized root-mean-squared residual (SRMR) values ≤ 0.08 would indicate acceptable fit and values ≤ 0.05 would indicate good fit.

To evaluate the direct effect of condition on the outcome and intermediate measures, we specified separate models for each measure to prevent loss of power due to a high number of parameters. For each measure, we first fitted a linear growth model including a latent intercept and a latent slope factor with factor loadings corresponding to the number of months between assessments (0, 1, 3, 5). Then, we regressed the intercept and linear slope on a condition variable that was dummy coded with 1 = intervention group and 0 = control group.

Indirect or mediation effects were only examined for intermediate outcomes that were significantly predicted by condition. To evaluate the indirect effects, we again specified separate models per intermediate outcome and outcome measure. For each combination of variables, we fitted similar linear growth models for the outcome measure and the intermediate outcome. Then, we specified the three direct regression paths, that is, (1) condition on the slope of the outcome, (2) condition on the slope of the intermediate outcome, and (3) the slope of the intermediate outcome on the slope of the outcome. Finally, we specified the indirect effect of condition on the slope of the outcome through the slope of the intermediate outcome.

#### Sensitivity analyses

We conducted two types of sensitivity analyses to check the robustness of our results. First, we reran analyses after excluding participants without YIMs from the intervention group. Second, we reran analyses after removing participants from the intervention group who received treatment with a low level of treatment fidelity (i.e., < 75% of steps performed).

## Results

### Participant flow

During the recruitment period, 290 youths started treatment at one of the participating treatment modalities, of which 182 at InConnection. Of these, 240 (83%) youths and their parents or caregivers (i.e., client system) were informed about the study by their case manager and asked if they consented to sharing their contact information with the independent research team, to which 148 (51%) of client systems agreed. Client systems were then approached by the research team, and 113 (39%) consented to participate in the study. Six client systems did not participate, resulting in 107 participating families of which 66 were in the intervention group. Consent and participation of individuals resulted in the final sample of *n* = 102 youths and *n* = 85 parents eligible for analyses. See Fig. 1 for the participant flow per treatment condition.

### Descriptive statistics

The means and standard deviations of all study variables are presented in Table 2. Correlations between study variables from the same informant are shown in Table 3.

**Table 2:** Descriptive statistics of study variables in both treatment conditions at all measurement occasions

	T1 – Baseline		T2–3 months after		T3–9 months after		T4–15 months after	
	*n*	*M* (*SD*) / %	*n*	*M* (*SD*) / %	*n*	*M* (*SD*) / %	*n*	*M* (*SD*) / %
Youth resilience (Y)	98	3.76 (0.53)	77	3.73 (0.56)	76	3.68 (0.63)	74	3.66 (0.62)
Care as usual	29	3.52 (0.48)	27	3.54 (0.68)	25	3.38 (0.76)	24	3.38 (0.68)
InConnection	69	3.87 (0.51)	50	3.82 (0.45)	51	3.82 (0.51)	50	3.80 (0.55)
Youth well-being (Y)	98	2.73 (1.17)	77	2.79 (1.17)	76	2.72 (1.24)	74	2.59 (1.24)
Care as usual	29	2.57 (1.28)	27	2.47 (1.36)	25	2.50 (1.26)	24	2.23 (1.25)
InConnection	69	2.79 (1.13)	50	2.96 (1.03)	51	2.82 (1.23)	50	2.76 (1.20)
Youth emotional/behavioral problems (Y)	98	0.68 (0.39)	77	0.62 (0.33)	76	0.62 (0.34)	74	0.62 (0.36)
Care as usual	29	0.69 (0.38)	27	0.68 (0.36)	25	0.72 (0.38)	24	0.67 (0.38)
InConnection	69	0.68 (0.40)	50	0.59 (0.31)	51	0.57 (0.30)	50	0.59 (0.35)
Youth emotional/behavioral problems (P)	85	1.00 (0.43)	63	0.92 (0.38)	73	0.91 (0.37)	66	0.87 (0.40)
Care as usual	31	0.91 (0.41)	22	0.89 (0.44)	28	0.92 (0.33)	26	0.95 (0.34)
InConnection	54	1.05 (0.44)	41	0.93 (0.35)	45	0.90 (0.39)	40	0.82 (0.44)
Risk of child unsafety (C)	105	5.07 (2.96)	72	4.78 (3.40)	77	4.23 (3.19)	51	3.43 (2.60)
Care as usual	34	4.65 (3.20)	25	5.00 (3.63)	26	4.31 (3.67)	15	2.67 (2.82)
InConnection	71	5.27 (2.84)	47	4.66 (3.30)	51	4.20 (2.96)	36	3.75 (4.00)
Out-of-home placements: Yes (Y)	-	-	76	14.5%	75	13.3%	74	12.2%
Care as usual	-	-	26	15.4%	25	8.0%	24	20.8%
InConnection	-	-	50	14.0%	50	16.0%	50	8.0%
Parent-child relationship quality (Y)	93	2.85 (0.67)	73	2.94 (0.64)	72	2.87 (0.61)	68	2.83 (0.70)
Care as usual	26	2.75 (0.72)	24	2.88 (0.71)	25	2.75 (0.60)	22	2.88 (0.62)
InConnection	67	2.89 (0.65)	49	2.96 (0.61)	47	2.93 (0.61)	46	2.80 (0.75)
Parent-child relationship quality (P)	85	3.11 (0.47)	63	3.01 (0.61)	73	3.16 (0.49)	67	3.08 (0.56)
Care as usual	31	3.09 (0.49)	22	3.11 (0.55)	28	3.12 (0.56)	26	3.06 (0.54)
InConnection	54	3.12 (0.46)	41	3.00 (0.64)	45	3.19 (0.46)	41	3.10 (0.57)
Parental resilience (P)	83	4.08 (0.52)	60	4.02 (0.49)	71	4.02 (0.49)	66	4.05 (0.55)
Care as usual	30	4.12 (0.54)	21	4.06 (0.50)	26	4.06 (0.50)	25	4.04 (0.54)
InConnection	53	4.06 (0.51)	39	4.00 (0.49)	45	3.99 (0.50)	41	4.06 (0.57)
Parental well-being (P)	63	2.93 (1.20)	52	2.97 (1.04)	71	2.95 (1.05)	63	2.72 (0.93)
Care as usual	29	2.98 (1.17)	21	3.04 (1.06)	26	2.96 (0.99)	25	2.78 (0.73)
InConnection	34	2.88 (1.24)	31	2.92 (1.04)	45	2.95 (1.10)	38	2.67 (1.05)
Parental empowerment (P)	80	3.90 (0.59)	59	3.85 (0.49)	70	3.91 (0.50)	65	3.91 (0.58)
Care as usual	29	3.84 (0.76)	21	3.77 (0.53)	26	3.80 (0.55)	25	3.71 (0.66)
InConnection	51	3.94 (0.48)	38	3.90 (0.46)	44	3.98 (0.45)	40	4.03 (0.49)
Positive parenting (P)	87	4.05 (0.60)	65	4.00 (0.69)	75	4.17 (0.59)	69	4.13 (0.55)
Care as usual	33	4.15 (0.60)	24	4.04 (0.49)	30	4.09 (0.62)	28	4.04 (0.51)
InConnection	54	3.99 (0.60)	41	3.98 (0.78)	45	4.23 (0.56)	41	4.20 (0.57)
Poor supervision (P)	87	2.34 (1.04)	64	2.34 (1.20)	75	2.39 (1.13)	66	2.42 (1.02)
Care as usual	33	2.26 (1.02)	24	2.73 (1.32)	30	2.67 (1.27)	28	2.59 (1.03)
InConnection	54	2.39 (1.05)	40	2.10 (1.08)	45	2.20 (1.01)	38	2.29 (1.01)
Inconsistent discipline (P)	86	2.71 (0.90)	65	2.58 (0.77)	75	2.50 (0.82)	66	2.55 (0.79)
Care as usual	32	2.69 (0.79)	24	2.54 (0.67)	30	2.46 (0.72)	28	2.58 (0.77)
InConnection	54	2.72 (0.96)	41	2.60 (0.82)	45	2.53 (0.88)	38	2.52 (0.81)
Social resourcefulness (Y)	98	2.11 (0.67)	76	2.24 (0.63)	75	2.13 (0.70)	73	2.16 (0.69)
Care as usual	29	2.02 (0.61)	26	2.22 (0.70)	25	1.99 (0.75)	23	2.18 (0.81)
InConnection	69	2.14 (0.70)	50	2.25 (2.59)	50	2.20 (0.67)	50	2.16 (0.64)
Shared-decision making (Y)	91	6.88 (2.47)	74	6.80 (2.11)	58	7.26 (2.29)	53	6.78 (2.27)
Care as usual	28	5.81 (3.10)	26	6.21 (2.67)	16	6.91 (2.45)	18	5.97 (2.95)
InConnection	63	7.35 (1.99)	48	7.11 (1.68)	42	7.39 (2.24)	35	7.20 (1.74)
Shared-decision making (P)	77	7.47 (1.98)	36	6.74 (1.87)	50	7.80 (1.85)	17	7.24 (1.59)
Care as usual	28	7.82 (2.09)	18	7.00 (1.89)	20	7.80 (2.01)	6	7.75 (1.89)
InConnection	49	7.27 (1.91)	18	6.47 (1.87)	30	7.80 (1.77)	11	6.95 (1.42)
Treatment motivation (Y)	96	3.58 (0.65)	74	3.56 (0.72)	57	3.49 (0.88)	54	3.53 (0.82)
Care as usual	29	3.74 (0.71)	26	3.76 (0.87)	16	3.42 (0.97)	18	3.65 (0.95)
InConnection	67	3.51 (0.61)	48	3.45 (0.61)	41	3.52 (0.85)	36	3.47 (0.76)

*Note.* Y = reported by youth; *P* = reported by parents; C = reported by case managers

**Table 3:** Correlations between Study Variables Within Informants at Baseline

	1.	2.	3.	4.	5.	6.	7.	8.	9.	10.	11.
1. Resilience	-	0.28*	− 0.08	− 0.11	0.29**	0.06	0.06	− 0.17	-	0.36**	-
2. Well-being	0.54**	-	− 0.16	0.31*	0.39**	0.26*	− 0.22	− 0.10	-	0.07	-
3. Emotional/behavioral problems	− 0.09	− 0.12	-	− 0.30**	0.03	− 0.09	0.26*	0.22*	-	− 0.01	-
4. Parent-child relationship quality	0.52**	0.35**	− 0.22*	-	0.37**	0.38**	− 0.21*	0.15	-	− 0.13	-
5. Parental empowerment	-	-	-	-	-	0.44**	− 0.03	0.06	-	0.23*	-
6. Positive parenting	-	-	-	-	-	-	0.01	0.01	-	− 0.01	-
7. Poor supervision	-	-	-	-	-	-	-	0.03	-	0.08	-
8. Inconsistent discipline	-	-	-	-	-	-	-	-	-	− 0.08	-
9. Social resourcefulness	0.16	0.07	0.09	0.23*	-	-	-	-	-	-	-
10. Shared-decision making	0.22*	0.31**	0.19	0.14	-	-	-	-	0.16	-	-
11. Treatment motivation	− 0.05	− 0.22*	− 0.11	0.06	-	-	-	-	− 0.05	− 0.34**	-

*Note.* The panels below the diagonal (bottom left) show the correlations between youth-reported variables, and the panels above the diagonal (top right) show the correlations between parent-reported variables. We did not report cross-informant correlations due to the small number of cases in which multiple informants from one family participated

* *p* < .05, ** *p* < .01

### Condition differences in study variables

In addition to equivalence on demographic factors between the treatment conditions (see Table 1), we also examined whether there were differences between the conditions on the outcome measures of this study at the first measurement occasion by examining the effect of condition on the intercepts. We found two significant differences, namely for youth-reported resilience and youth-reported shared-decision making, *p* = .002, and *p* = .028, respectively. In both cases, youths reported higher levels in the intervention group than in the control group. No differences were found for parent-reported and case manager-reported outcomes (see Table 4).

**Table 4:** Results of the latent growth model analyses comparing the treatment effectiveness of inconnection to care as usual

	Intercept			Slope			Model fit indices		
	*M*	σ^2^	*b* (*SE*)	*M*	σ^2^	*b* (*SE*)	CFI	RMSEA	SRMR
Youth resilience (Y)	3.51**	0.15**		-0.03	0.01**		1.000	0.000	0.074
Effect of condition			0.35 (0.11)**			0.02 (0.03)			
Youth well-being (Y)	2.52**	0.54**		-0.04	0.02		1.000	0.000	0.031
Effect of condition			0.37 (0.24)			0.02 (0.06)			
Youth E/B problems (Y)^1^	0.70**	0.09**		-0.01	0.00		1.000	0.000	0.044
Effect of condition			-0.07 (0.08)			-0.01 (0.01)			
Youth E/B problems (P)	0.89**	0.12**		0.00	0.00		0.958	0.076	0.083
Effect of condition			0.13 (0.10)			-0.05 (0.02)*			
Risk of child unsafety (C)	5.20**	7.31**		-0.32**	0.10		0.972	0.064	0.079
Effect of condition			-0.45 (0.66)			-0.02 (0.14)			
Out-of-home placements (Y)^1^	0.14	0.05		0.01	0.00		0.851	0.075	0.095
Effect of condition			0.04 (0.09)			-0.02 (0.02)			
Parent-child relationship quality (Y)	2.77**	0.32**		0.02	0.00		0.972	0.073	0.077
Effect of condition			0.16 (0.15)			-0.02 (0.03)			
Parent-child relationship quality (P)^1^	3.16**	0.15**		-0.01	0.00		0.921	0.093	0.212
Effect of condition			-0.07 (0.12)			0.03 (0.03)			
Parental resilience (P)	4.12**	0.18**		-0.03	0.00		0.999	0.015	0.117
Effect of condition			-0.10 (0.11)			0.03 (0.02)			
Parental well-being (P)	3.02**	1.04**		-0.04	0.02		1.000	0.000	0.038
Effect of condition			-0.03 (0.28)			-0.01 (0.06)			
Parental empowerment (P)^1^	3.87**	0.14**		-0.02	0.00		0.989	0.039	0.064
Effect of condition			0.02 (0.13)			0.05 (0.03)			
Positive parenting (P)	4.18**	0.27**		-0.03	0.01*		1.000	0.000	0.070
Effect of condition			-0.16 (0.15)			0.09 (0.03)**			
Poor supervision (P)	2.40**	0.75**		0.05	0.01		0.978	0.067	0.075
Effect of condition			-0.04 (0.27)			-0.06 (0.04)			
Inconsistent discipline (P)	2.65**	0.44**		-0.03	0.01		1.000	0.000	0.061
Effect of condition			0.03 (0.18)			-0.02 (0.04)			
Social resourcefulness (Y)^1^	2.07**	0.30**		0.01	0.00		0.991	0.030	0.127
Effect of condition			0.11 (0.13)			0.00 (0.03)			
Shared-decision making (Y)^1^	6.04**	2.00**		0.01	0.00		1.000	0.000	0.079
Effect of condition			1.23 (0.56)*			-0.03 (0.13)			
Shared-decision making (P)^1,2^	7.71**	2.60*		-0.07	0.00		0.828	0.118	0.058
Effect of condition			-0.50 (0.46)			0.28 (0.19)			
Treatment motivation (Y)	3.76**	0.32**		-0.01	0.02*		1.000	0.000	0.072
Effect of condition			-0.25 (0.15)			0.01 (0.15)			

*Note. M* = mean of intercept or slope; σ^2^ = variance of intercept or slope; *b* (*SE*) = regression coefficient (and standard error) of condition on intercept or slope; CFI = comparative fit index; RMSEA = root mean square error of approximation; SRMR = standardized root mean square residual; Y = reported by youth; *P* = reported by parents; C = reported by case managers; Youth E/B problems = Youth emotional/behavioral problems. Control group is the reference category (0).

^1^ Due to negative slope variance, we constrained the slope variance (> 0). The negative slope variance indicates that there is no variance to be explained by condition. Thus, we could not reliably estimate the influence of condition on the slope in these models, and any significant effects are ignored.

^2^ Due to the low number of parents at T4, we estimated this model using only T1, T2 and T3.

* *p* < .05, ** *p* < .01

### Intervention effects

#### Direct effects on primary and secondary outcomes

**Fig. 2:**
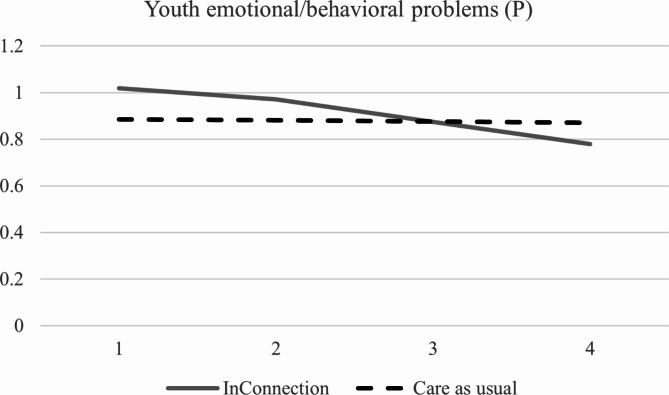
Graph with results from the latent growth model for parent-reported youth emotional/behavioral problems *Note*: This figure shows the significant treatment effect of InConnection compared to care as usual on parent-reported youth emotional/behavioral problems, *p* = .013. InConnection: *b* = -0.05; care as usual: *b* = 0.00.

**Fig. 3:**
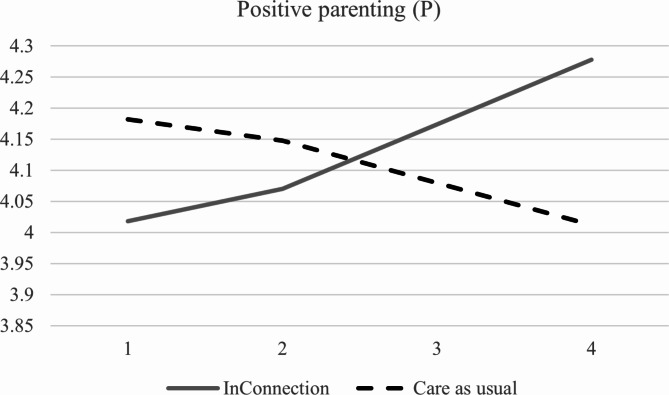
Graph with results from the latent growth model for parent-reported positive parenting *Note*: This figure shows the significant treatment effect of InConnection compared to care as usual on parent-reported positive parenting, *p* = .003. InConnection: *b* = 0.05; care as usual: *b* = -0.03.

To evaluate the direct effects on the primary and secondary outcomes, we conducted separate models in which the linear slope of an outcome measure was regressed on the condition variable. Results indicated few significant treatment effects. Overall, youths and parents did not experience changes over time in any of the outcome variables, indicated by insignificant slope values. Case managers from both conditions, however, did report decreases in child unsafety, *p* < .001. When looking at the effect of condition on the slopes, which would indicate differences in effectiveness between conditions, we did not find any treatment effects on self-reported youth outcomes (*p*s > 0.229) nor for child unsafety reported by case managers (*p* = .901). However, we found a significant treatment effect on parent-reported youth emotional and behavioral problems (see Fig. 2), *b* = -0.05, *SE* = 0.02, *p* = .013, β = -0.76. Concerning parents’ self-reported outcomes, we found one significant treatment effect on positive parenting (see Fig. 3), *b* = 0.09, *SE* = 0.03, *p* = .003, β = 0.42. Thus, on average, parents in the intervention condition reported improvements in youths’ emotional and behavioral problems and their own positive parenting over time, whereas control parents did not experience changes in these outcomes. No treatment effects were found for the other parent-reported outcome measures (*p*s > 0.112). Most models had acceptable to good fit. See Table 4 for detailed model results.

#### Direct effects on intermediate outcomes

To examine the treatment effects on the four intermediate outcomes, we conducted similar separate models to the analyses presented above. Youths and parents did not report any significant changes in the intermediate outcomes over time. We also did not find any effect of condition on change for youths nor parents, *p*s > 0.850 and *p* = .568, respectively. Therefore, we did not perform mediation analyses. Most models had acceptable to good fit. See Table 4 for detailed results of all models.

#### Sensitivity analyses

We conducted two types of sensitivity analyses to check the robustness of our results concerning the treatment effects on the primary, secondary and intermediate outcomes. That is, we reran analyses after excluding *n* = 41 cases from the intervention group without YIMs, and after excluding *n* = 40 cases with low levels of treatment fidelity. The results (see Tables 1 and 2 of the Appendix) were similar to those from the initial analyses, giving us confidence in the accuracy of our results. We found significant treatment effects in both sensitivity analyses on positive parenting (*p* = .020, β = 0.41, and *p* = .001, β = 0.75, for sensitivity analyses excluding cases without YIMs, and cases with low treatment fidelity, respectively). For parent-reported youth emotional and behavioral problems, we found one significant and one marginally significant treatment effect. The treatment effect on parent-reported youth emotional and behavioral problems was significant in the analyses excluding cases without YIMs (*p* = .004, β = -0.52), and marginally significant in the analyses excluding cases with low treatment fidelity (*p* = .050, β = -0.69). None of the other sensitivity analyses yielded significant results.

## Discussion

This study investigated the effectiveness of the InConnection approach, a multidisciplinary treatment for youths with mental health needs from multi-problem families that utilizes the youth’s social network by collaborating with a YIM. Results showed that, in general, families neither reported improvements nor declines in their functioning over the study period. Yet, case managers in both conditions reported decreases in child unsafety, suggesting that both treatment conditions may have decreased the very serious problem of the risk of child maltreatment, but not other problems, although this decrease could be an effect of time rather than a treatment effect. The InConnection approach did not outperform care as usual on most outcome variables, including the primary outcome of youth resilience. Yet, two treatment effects should be noted. That is, parents in the InConnection condition reported reductions in their children’s emotional and behavioral problems, and improvements in their own positive parenting, whereas control parents did not report any changes. We found no effects on the intermediate outcomes, which might implicate that social resourcefulness, shared-decision making and treatment motivation may not be working mechanisms specific to the InConnection approach.

The results failed to confirm our hypotheses that the InConnection approach would yield greater effects than care as usual on self-reported youth functioning, including resilience, although treatment effects were found on parent-reported youth emotional and behavioral problems. Congruent with this finding, recent meta-analyses also demonstrated that psychological care in general [74], and treatment programs for youths with multiple problems specifically [5], are generally not effective in improving youth functioning. Yet, youth functioning in both treatment conditions was not reduced, which demonstrates that neither of the two treatment conditions had harmful effects. The stability in functioning could potentially be a result of successful treatment. That is, due to the complexity and seriousness of problems in this sample [1, 2], we may expect declines in functioning had these families not received treatment as mental health problems have the potential to evolve into chronic disorders leading to subsequent adverse consequences [75]. However, an experimental design with a group that does not receive treatment is needed to confirm or reject this hypothesis.

The absence of treatment effects on (self-reported) youth functioning for InConnection may be attributed to the relatively low treatment fidelity of this approach [76], potentially leading to unintended similarities between treatment conditions. Yet, our sensitivity analysis, excluding the low fidelity cases, showed similar results, giving us more confidence in the accuracy of the results.

Interestingly, the two treatment effects that we found were both reported by parents, while the two unique elements of the InConnection approach are aimed at youth. That is, the integration of care is primarily focused on youth, and the YIM is positioned to support youths [25]. Yet, the integration of care also allows parents to receive different forms of treatment, including elements of parenting programs. Improving parenting is a beneficial pathway to enhance the well-being of their children. Notably, treatment programs that positively impact parents, such as parenting programs, have been shown to result in improvements in their children [77, 78]. It is possible that it takes longer for youths to benefit from the InConnection approach (i.e., sleeper effects) and that the study duration was too short to detect these improvements [79], especially since treatment had not ended yet in most cases in the current study. Future research should aim to include follow-up assessments to examine whether youths benefit from InConnection in the long run, and whether these treatment effects are mediated by treatment effects on parents. Situational specificity may be another explanation for why parents in the InConnection condition reported improvements in youths’ emotional and behavioral problems, whereas youths themselves did not. That is, individuals may exhibit varying behaviors in different situations, leading to inconsistencies in reports across informants, and highlighting the importance of using multi-informant data [80]. As parents mostly report about their children’s behavior at home, whereas youths themselves report about their behavior across different contexts, this could suggest that InConnection improved youths’ behavior at home, but less in other contexts.

Another explanation for the limited treatment effects may be the Covid-19 pandemic, which started approximately one year after the start of this quasi-experimental study. This potentially influenced treatment effectiveness in several ways. First, the pandemic potentially extended treatment durations as appointments were postponed or done online due to lockdowns and social distancing measures. In fact, only one-third of treatments were completed by the final assessment (i.e., 15 months after starting treatment), whereas treatments in both conditions were supposed to last six to twelve months. Second, the pandemic has likely impacted the availability and stability of professional support due to social distancing measures and an increase in sick leave among professionals as a result of illness and increased stress. Similarly, the imposed measures potentially also influenced the availability of other support systems [81], including YIMs. Third, both youths and parents have likely been directly affected by the imposed measures [82, 83], such as school closure and working from home, as these measures caused shifts in family routines, daily functioning and social connectedness [81]. Studies indeed demonstrated that the pandemic negatively affected youths’ and parents’ well-being [84–88]. Youths may have been particularly affected by the pandemic, since the imposed measures impacted social activities, which are particularly important during adolescence [82, 83]. Thus, the Covid-19 pandemic has potentially negatively affected overall treatment effects or canceled out the positive treatment effects, especially in youth.

We did not find any effects over time on the intermediate outcomes, suggesting that social resourcefulness, shared-decision making and treatment motivation may not be working mechanisms specific to the InConnection approach. InConnection youths reported higher levels of shared-decision making throughout treatment compared to control youths, and this difference was already present at the first measurement occasion. As shared-decision making was assessed as the level of agreement with the professional on therapy goals and tasks, alternative to indicating a lack of random allocation to conditions, the effect might also reflect that suggesting to use InConnection as treatment and inviting youths to find and nominate a YIM provides these youths with more opportunities to experience shared-decision making during the intake phase. Notably, the initial high levels of perceived shared-decision making in InConneciton youths reduced the chance of finding an intervention effect. Therefore, both true pre-test assessments and a randomized controlled trial are warranted to examine shared-decision making and other mediators of InConnection, while controlling for selection effects and other potential biases [89].

### Limitations and strengths

The current study has several limitations. First, our sample size was smaller than initially planned [11], as fewer families started treatment at one of the participating organizations than expected due to the dissolution of one organization (Juzt) and limited budget from the municipalities for youth care. The smaller sample size limited the possibility to examine the impact of moderators (e.g., demographic factors) and predictors (e.g., YIM relationship quality) of intervention effects. Additionally, due to the small sample, we had to run 18 separate analyses for the different measures. This multiplicity or multiple testing may have led to finding significant results solely by chance [90]. Therefore, the few significant results found in this study should be interpreted with caution. Yet, Streiner and Norman [90] suggest not to correct for multiple testing if hypotheses are formulated, as we did in this study, since a priori hypotheses decrease the probability that results are due to chance. Second, this study has limitations typical of a quasi-experimental design, such as selection effects [89]. That is, participant inclusion was based on multiple criteria that potentially differed per case, such as the severity of problems, and condition assignment was based on factors such as availability and preferences. A meta-analysis demonstrated that the self-selection in quasi-experimental studies could lead to an underestimation of intervention effects [91]. In this study, we demonstrated that the families in both conditions did not significantly differ in terms of demographics, yet youths in the treatment group reported higher levels of resilience and shared-decision making at the first measurement occasion, indicating that the two groups were not completely comparable. Third, the duration of the study was not long enough to examine long-term effects. Although the fourth measurement occasion was meant as a follow-up assessment, most treatments were not completed yet. This limits our understanding of the effectiveness of the InConnection approach at and after completion of treatment. Fourth, the broad age range in our study (10–23 years) may have led to variations in the treatment techniques offered across families. However, this age range aligns with the families targeted by the interventions, enabling the assessment of program effectiveness in real-world settings, where such age ranges are commonly encountered. Moreover, a meta-analysis demonstrated that the effectiveness of YIM programs does not vary by age [10]. Fifth, families in both conditions could use other forms of care during the study, as we examined the real-world effectiveness of the InConnection approach. Yet, since this possibility was equally present in both conditions, it likely did not impact the comparability between groups.

This study also has several strengths. First, the study was conducted under real-life circumstances, thus testing the effectiveness rather than the efficacy of the InConnection approach, which optimizes the ecological validity, and improves the generalizability to other real-life settings. Second, this study compared different active treatment conditions, which is considered to be a particularly rigorous standard of comparison [5, 92, 93].

### Future research

More research on the effectiveness of the InConnection approach is warranted, due to the impact of the problems of these families on their lives and society [1, 2], and the limited positive treatment effects of InConnection, despite the suboptimal conditions of this study. In general, there is a need for more robust, high-quality research examining the effectiveness of InConnection. Randomized-controlled trials (RCT) are considered the golden standard of intervention research because randomization reduces selection bias [89]. Therefore, future studies should aim to conduct RCTs with sample sizes that are sufficiently large to advance our understanding of for whom and under what circumstances these types of care programs work by examining moderators of treatment effects [32]. For example, research could investigate whether some treatment techniques used in InConnection work better than others. That is, behavioral treatments, including cognitive-behavioral therapy, have been found to be more effective for improving a wide range of psychological problems in other at-risk populations [94–96] than non-behavioral and multisystem treatment approaches [5]. Since families in this study were offered various types of treatments, including interventions with limited evidence base, the InConnection approach could potentially be enhanced by selecting evidence-based treatment elements and techniques.

To further our knowledge on the effectiveness of InConnection, it is valuable to include follow-up assessments in future research to investigate potential sleeper effects [79] and examine whether youths benefit from InConnection in the long run. Additionally, follow-up studies can examine whether the potential treatment effects on youths are mediated by treatment effects on parents. Future research could also investigate whether the treatment offered in the InConnection approach meets families’ needs, which is an important element contributing to effectiveness according to youths and parents from multi-problem families [21]. Although the InConnection manual states that treatment elements should be selected according to families’ needs [25], no studies have examined yet whether this is the case and to what extent this contributes to treatment effectiveness.

## Conclusion

In sum, the InConnection approach outperforms care as usual only in two parent-reported outcomes: youth emotional and behavioral problems and positive parenting. Although the positive effects compared to care as usual are small in number, the absence of negative effects and the positive views families have of this treatment [97] suggest that the InConnection approach can be a valuable treatment for multi-problem families, especially until more effective treatment programs or elements have been developed, which could be used to enhance or replace existing treatments.

### Electronic supplementary material

Below is the link to the electronic supplementary material.

Supplementary Material 1

## Data Availability

The dataset analyzed during the current study is accessible by all authors. Access was also granted to students or research assistants who assisted in data collection for the duration of their research project membership, after signing a confidentiality agreement. The dataset is not publicly available due to sensitivity and confidentiality of data, but is available from the corresponding author on reasonable request after completion of the research project.
