# Supplementary material for: Effectiveness of a multidisciplinary treatment with youth-initiated mentoring for youths with mental health needs from multi-problem families: a quasi-experimental study
Source: BMC Public Health. 2024 Jan 2;24:1. doi: 10.1186/s12889-023-17506-6 (PMC10759347; doi:10.1186/s12889-023-17506-6)
Supplement: Supplementary file 1 — Supplementary Material 1 [file 12889_2023_17506_MOESM1_ESM.docx]

**Appendix**

**Table S1**

*Sensitivity Results of the Latent Growth Model Analyses Comparing the Treatment Effectiveness of InConnection to Care as Usual for Youth with a YIM*

|  | Intercept | | | Slope | | | Model fit indices | | |
| --- | --- | --- | --- | --- | --- | --- | --- | --- | --- |
|  | *M* | σ^2^ | *b* (*SE*) | *M* | σ^2^ | *b* (*SE*) | CFI | RMSEA | SRMR |
| Youth resilience (Y) | 3.50** | 0.16** |  | -0.02 | 0.01** |  | 0.975 | 0.077 | 0.126 |
| Effect of condition |  |  | 0.43 (0.12)** |  |  | -0.02 (0.03) |  |  |  |
| Youth well-being (Y) | 2.51** | 0.67** |  | -0.04 | 0.02 |  | 1.000 | 0.000 | 0.081 |
| Effect of condition |  |  | 0.45 (0.26) |  |  | -0.02 (0.06) |  |  |  |
| Youth E/B problems (Y)^1^ | 0.70** | 0.10** |  | -0.01 | 0.00 |  | 1.000 | 0.000 | 0.054 |
| Effect of condition |  |  | -0.04 (0.09) |  |  | -0.01 (0.01) |  |  |  |
| Youth E/B problems (P) | 0.89** | 0.14** |  | 0.00 | 0.00 |  | 0.985 | 0.052 | 0.141 |
| Effect of condition |  |  | 0.11 (0.11) |  |  | -0.06 (0.02)** |  |  |  |
| Risk of child unsafety (C) | 5.06** | 5.42** |  | -0.31** | 0.07 |  | 0.971 | 0.064 | 0.091 |
| Effect of condition |  |  | -0.12 (0.99) |  |  | 0.15 (0.18) |  |  |  |
| Out-of-home placements (Y)^1^ | 0.12 | 0.01 |  | 0.01 | 0.00 |  | 1.000 | 0.000 | 0.084 |
| Effect of condition |  |  | 0.01 (0.09) |  |  | -0.02 (0.03) |  |  |  |
| Parent-child relationship quality (Y) | 2.77** | 0.29** |  | 0.02 | 0.00 |  | 0.977 | 0.061 | 0.085 |
| Effect of condition |  |  | 0.20 (0.16) |  |  | -0.01 (0.03) |  |  |  |
| Parent-child relationship quality (P)^1^ | 3.17** | 0.17** |  | -0.01 | 0.00 |  | 0.969 | 0.062 | 0.218 |
| Effect of condition |  |  | -0.05 (0.14) |  |  | 0.05 (0.03) |  |  |  |
| Parental resilience (P) | 4.12** | 0.17** |  | -0.03 | 0.00 |  | 1.000 | 0.000 | 0.080 |
| Effect of condition |  |  | -0.08 (0.11) |  |  | 0.03 (0.02) |  |  |  |
| Parental well-being (P) | 3.03** | 1.16** |  | -0.04 | 0.02 |  | 1.000 | 0.000 | 0.065 |
| Effect of condition |  |  | 0.04 (0.34) |  |  | -0.04 (0.07) |  |  |  |
| Parental empowerment (P)^1^ | 3.87** | 0.12* |  | -0.02 | 0.00 |  | 1.000 | 0.000 | 0.091 |
| Effect of condition |  |  | 0.00 (0.13) |  |  | 0.05 (0.03) |  |  |  |
| Positive parenting (P) | 4.18** | 0.26** |  | -0.03 | 0.01 |  | 1.000 | 0.000 | 0.065 |
| Effect of condition |  |  | -0.14 (0.16) |  |  | 0.08 (0.03)* |  |  |  |
| Poor supervision (P) | 2.41** | 0.62** |  | 0.05 | 0.00 |  | 0.987 | 0.053 | 0.094 |
| Effect of condition |  |  | -0.36 (0.29) |  |  | -0.01 (0.04) |  |  |  |
| Inconsistent discipline (P) | 2.65** | 0.37** |  | -0.03 | 0.01 |  | 1.000 | 0.000 | 0.079 |
| Effect of condition |  |  | 0.15 (0.19) |  |  | -0.03 (0.05) |  |  |  |
| Social resourcefulness (Y) | 2.06** | 0.25** |  | 0.01 | 0.00 |  | 0.988 | 0.032 | 0.127 |
| Effect of condition |  |  | 0.06 (0.14) |  |  | 0.00 (0.03) |  |  |  |
| Shared-decision making (Y) | 6.04** | 3.00** |  | -0.03 | 0.15 |  | 1.000 | 0.000 | 0.123 |
| Effect of condition |  |  | 1.55 (0.57)** |  |  | -0.05 (0.14) |  |  |  |
| Shared-decision making (P)^2^ | 7.62** | 1.55 |  | 0.00 | 0.07 |  | 0.113 | 0.226 | 0.082 |
| Effect of condition |  |  | 0.08 (0.45) |  |  | 0.10 (0.22) |  |  |  |
| Treatment motivation (Y) | 3.76** | 0.37** |  | -0.01 | 0.03* |  | 1.000 | 0.000 | 0.058 |
| Effect of condition |  |  | -0.29 (0.16) |  |  | 0.04 (0.06) |  |  |  |

*Note.* *M* = mean of intercept or slope; σ^2^ = variance of intercept or slope; *b* (*SE*) = regression coefficient (and standard error) of condition on intercept or slope; CFI = comparative fit index; RMSEA = root mean square error of approximation; SRMR = standardized root mean square residual; Y = reported by youth; P = reported by parents; C = reported by case managers; Youth E/B problems = Youth emotional/behavioral problems. Control group is the reference category (0).

^1^ Due to negative slope variance, we constrained the slope variance (>0). The negative slope variance indicates that there is no variance to be explained by condition. Thus, we could not reliably estimate the influence of condition on the slope in these models, and any significant effects are ignored.

^2^ Due to the low number of parents at T4, we estimated this model using only T1, T2 and T3.

* *p* < .05, ** *p* < .01

**Table S2**

*Sensitivity Results of the Latent Growth Model Analyses Comparing the Treatment Effectiveness of InConnection to Care as Usual for Cases with High Treatment Fidelity*

|  | Intercept | | | Slope | | | Model fit indices | | |
| --- | --- | --- | --- | --- | --- | --- | --- | --- | --- |
|  | *M* | σ^2^ | *b* (*SE*) | *M* | σ^2^ | *b* (*SE*) | CFI | RMSEA | SRMR |
| Youth resilience (Y) | 3.51** | 0.17** |  | -0.02 | 0.01** |  | 0.943 | 0.121 | 0.192 |
| Effect of condition |  |  | 0.36 (0.13)** |  |  | 0.04 (0.03) |  |  |  |
| Youth well-being (Y) | 2.51** | 0.71** |  | -0.04 | 0.01 |  | 0.916 | 0.096 | 0.123 |
| Effect of condition |  |  | 0.28 (0.28) |  |  | 0.07 (0.06) |  |  |  |
| Youth E/B problems (Y)^1^ | 0.69** | 0.10** |  | 0.00 | 0.00 |  | 1.000 | 0.000 | 0.061 |
| Effect of condition |  |  | -0.06 (0.09) |  |  | -0.02 (0.01) |  |  |  |
| Youth E/B problems (P) | 0.88** | 0.15** |  | 0.00 | 0.00 |  | 0.976 | 0.064 | 0.116 |
| Effect of condition |  |  | 0.08 (0.12) |  |  | -0.04 (0.02) |  |  |  |
| Risk of child unsafety (C)^1^ | 5.02** | 7.50** |  | -0.35** | 0.00 |  | 1.000 | 0.000 | 0.083 |
| Effect of condition |  |  | -0.25 (0.72) |  |  | 0.01 (0.15) |  |  |  |
| Out-of-home placements (Y)^1^ | 0.13 | -0.03 |  | 0.01 | 0.00 |  | 1.000 | 0.000 | 0.108 |
| Effect of condition |  |  | -0.02 (0.10) |  |  | 0.00 (0.03) |  |  |  |
| Parent-child relationship quality (Y)^1^ | 2.76** | 0.29** |  | 0.02 | 0.00 |  | 1.000 | 0.000 | 0.122 |
| Effect of condition |  |  | 0.09 (0.17) |  |  | 0.01 (0.03) |  |  |  |
| Parent-child relationship quality (P)^1^ | 3.16** | 0.16** |  | -0.01 | 0.00 |  | 1.000 | 0.000 | 0.166 |
| Effect of condition |  |  | 0.02 (0.13) |  |  | 0.03 (0.03) |  |  |  |
| Parental resilience (P) | 4.12** | 0.17** |  | -0.03 | 0.00 |  | 1.000 | 0.000 | 0.120 |
| Effect of condition |  |  | -0.05 (0.12) |  |  | 0.03 (0.02) |  |  |  |
| Parental well-being (P) | 3.02** | 1.08** |  | -0.03 | 0.01 |  | 1.000 | 0.000 | 0.058 |
| Effect of condition |  |  | 0.21 (0.34) |  |  | -0.09 (0.08) |  |  |  |
| Parental empowerment (P)^1^ | 3.88** | 1.14* |  | -0.02 | 0.00 |  | 0.938 | 0.108 | 0.197 |
| Effect of condition |  |  | 0.11 (0.14) |  |  | 0.06 (0.02) |  |  |  |
| Positive parenting (P) | 4.17** | 0.28* |  | -0.03 | 0.00 |  | 1.000 | 0.000 | 0.060 |
| Effect of condition |  |  | -0.19 (0.17) |  |  | 0.11 (0.03)** |  |  |  |
| Poor supervision (P) | 2.40** | 0.74** |  | 0.05 | 0.02 |  | 0.921 | 0.132 | 0.115 |
| Effect of condition |  |  | -0.30 (0.32) |  |  | -0.03 (0.06) |  |  |  |
| Inconsistent discipline (P) | 2.66** | 0.36** |  | -0.03 | 0.01 |  | 1.000 | 0.000 | 0.065 |
| Effect of condition |  |  | 0.01 (0.20) |  |  | 0.00 (0.06) |  |  |  |
| Social resourcefulness (Y) | 2.07** | 0.24** |  | 0.01 | 0.00 |  | 0.978 | 0.044 | 0.131 |
| Effect of condition |  |  | 0.20 (0.16) |  |  | -0.01 (0.04) |  |  |  |
| Shared-decision making (Y) | 6.12** | 2.80** |  | 0.00 | 0.03 |  | 0.968 | 0.059 | 0.070 |
| Effect of condition |  |  | 1.07 (0.61) |  |  | 0.08 (0.15) |  |  |  |
| Shared-decision making (P)^1,2^ | 7.72** | 2.92* |  | -0.09 | 0.00 |  | 0.588 | 0.208 | 0.087 |
| Effect of condition |  |  | 0.34 (0.57) |  |  | 0.20 (0.23) |  |  |  |
| Treatment motivation (Y) | 3.76** | 0.32** |  | -0.02 | 0.03* |  | 1.000 | 0.000 | 0.082 |
| Effect of condition |  |  | -0.29 (0.15) |  |  | 0.01 (0.06) |  |  |  |

*Note.* *M* = mean of intercept or slope; σ^2^ = variance of intercept or slope; *b* (*SE*) = regression coefficient (and standard error) of condition on intercept or slope; CFI = comparative fit index; RMSEA = root mean square error of approximation; SRMR = standardized root mean square residual; Y = reported by youth; P = reported by parents; C = reported by case managers; Youth E/B problems = Youth emotional/behavioral problems. Control group is the reference category (0).

^1^ Due to negative slope variance, we constrained the slope variance (>0). The negative slope variance indicates that there is no variance to be explained by condition. Thus, we could not reliably estimate the influence of condition on the slope in these models, and any significant effects are ignored.

^2^ Due to the low number of parents at T4, we estimated this model using only T1, T2 and T3.

* *p* < .05, ** *p* < .01
